# Real-world effectiveness of second-line Afatinib versus chemotherapy for the treatment of advanced lung squamous cell carcinoma in immunotherapy-naïve patients

**DOI:** 10.1186/s12885-021-08920-3

**Published:** 2021-11-15

**Authors:** You-Yi Chen, Shih-Chieh Chang, Cheng-Yu Chang, Chun-Fu Chang, Yi-Chun Lai, Yu-Feng Wei, Chung-Yu Chen

**Affiliations:** 1 Division of Pulmonary and Critical Care Medicine, Department of Internal Medicine, National Taiwan University Hospital Yunlin Branch, Yunlin Count Douliu City, Taiwan, Republic of China; 2 College of Medicine, National Taiwan University, Taipei, Taiwan; 3 Division of Chest Medicine, Department of Internal Medicine, National Yang Ming Chiao Tung University Hospital, Yilan County, Taiwan; 4 Division of Pulmonary Medicine, Department of Internal Medicine, Far Eastern Memorial Hospital, and Department of Nursing, Cardinal Tien College of Healthcare and Management, New Taipei City, Taiwan; 5 Department of Internal Medicine, E-Da Cancer Hospital, Kaohsiung, Taiwan; 6 School of Medicine for International Students, College of Medicine, and Institute of Biotechnology and Chemical Engineering, I-Shou University, Kaohsiung, Taiwan

**Keywords:** Afatinib, Lung squamous cell carcinoma, Second-line treatment

## Abstract

**Background:**

Limited treatment options exist for relapsed advanced lung squamous cell carcinoma (SCC), leading to poor outcomes compared with adenocarcinoma. This study aimed to investigate the efficacy of second-line afatinib versus chemotherapy in patients with advanced lung SCC who progressed after first-line chemotherapy.

**Methods:**

In this retrospective, multisite cohort study, we recruited patients with initial locally advanced or metastatic lung SCC from four institutes in Taiwan between June 2014 and October 2020. The primary endpoint of this study was progression-free survival (PFS), and the secondary endpoints were the objective response rate (ORR), disease control rate (DCR), and overall survival (OS).

**Results:**

The present study enrolled 108 patients: 19 received second-line afatinib, and 89 received second-line chemotherapy. The median ages were 71 and 67 years, respectively. PFS was significantly longer among patients who received afatinib than among those who received chemotherapy (median 4.7 months [95% confidence interval (CI), 0.1–7.5] vs. 2.6 months [95% CI, 0.9–6.7]; hazard ratio (HR) 0.53 [95% CI 0.32–0.88], *p* = 0.013). Compared with the chemotherapy group, OS was longer in the afatinib group but did not reach significance (median 16.0 months [95% CI, 6.1–22.0] vs. 12.3 months [6.2–33.9]; HR 0.65 [95% CI 0.38–1.11], *p* = 0.112).

**Conclusions:**

Afatinib offered a longer PFS and comparable OS to chemotherapy in advanced lung SCC patients in a real-world setting, it may be considered as a 2nd line alternative treatment choice for immunotherapy unfit advanced lung SCC patients.

**Supplementary Information:**

The online version contains supplementary material available at 10.1186/s12885-021-08920-3.

## Background

Lung cancer is the leading cause of cancer-related death worldwide, and non-small-cell lung cancer (NSCLC) accounts for 80–85% of cases [1]. Among NSCLC, lung squamous cell carcinoma (SCC) is the second most common histology type, accounting for 20–30% of cases [2, 3]. The treatment of lung adenocarcinoma has evolved considerably due to the emergence of specifically targeted therapies against identified molecular drivers, such as epidermal growth factor receptor (EGFR), anaplastic lymphoma kinase (ALK), c-ros oncogene 1 (ROS1), and murine sarcoma viral oncogene homolog B (BRAF). By contrast, advancements in the targeted treatment of SCC have remained limited due to high molecular heterogeneity [4, 5]. Mutations are infrequently observed in the genes encoding members of the ErbB family of receptor tyrosine kinases, and targeted therapy is available for these cases [6]; however, the nature of mutations identified in lung SCC differs considerably from those identified in lung adenocarcinoma, resulting in sizable differences in treatment efficacy [7]. For advanced NSCLC patients with no targetable molecular drivers, platinum-based doublet chemotherapy has long been the standard first-line treatment, with good performance status, until the recent introduction of immunotherapy [8, 9].

In the recent past, immunotherapy targeting immune checkpoint proteins has been the most promising novel cancer treatment, and the introduction of immune checkpoint inhibitors (ICIs) into routine clinical practice has improved outcomes in patients with lung SCC. ICIs play a prominent role as first-line therapy, and common regimens include pembrolizumab plus carboplatin and paclitaxel/nab-paclitaxel, regardless of programmed death-ligand 1 (PD-L1) level [10, 11]; pembrolizumab monotherapy [11, 12] or nivolumab plus ipilimumab [13] in patients with a PD-L1 tumor proportion score (TPS) ≥ 1%; and atezolizumab monotherapy or cemiplimab monotherapy in patients with high PD-L1 expression, [14, 15] all of which have provided promising survival benefits.

Although ICIs have altered the first-line treatment paradigms for patients with lung SCC, few treatment options are available for advanced SCC of the lung after the failure of first-line platinum-based doublet chemotherapy or immunotherapy-based treatment. Docetaxel is generally considered to be a standard second-line treatment when patients progress after treatment with platinum-based chemotherapy [11, 16], and the addition of ramucirumab to docetaxel may further improve survival but increase toxicity [17]. Other chemotherapy options including gemcitabine monotherapy or platinum-based chemotherapy (if not included as first-line treatment). The only approved chemotherapy-free targeted therapy for second-line treatment is the pan-human epidermal growth factor receptor (HER) family tyrosine kinase inhibitor (TKI), afatinib, which demonstrated significant improved progression-free survival (PFS; median 2.4 vs. 1.9 months) and overall survival (OS; median 7.9 vs. 6.8 months) during the LUX-Lung 8 trial [18]. However, the benefits of afatinib were established based on comparisons with erlotinib rather than relative to chemotherapy. Therefore, the use of afatinib for second- or third-line therapy is currently recognized by the European Society of Medical Oncology (ESMO) but is not recommended by the US National Comprehensive Cancer Network (NCCN) Clinical Practice Guidelines in Oncology. Clinical evidence regarding the efficacy of afatinib versus chemotherapy for relapsed lung SCC remains lacking. In this study, we aimed to explore the real-world efficacy and safety of afatinib compared with chemotherapy.

## Methods

The present study was performed as a retrospective, observational, multicenter study. Our aim was to evaluate the treatment outcomes and safety of afatinib versus chemotherapy in patients with advanced SCC who progressed after first-line chemotherapy in a real-world setting. We retrospectively recruited patients with initial locally advanced or metastatic lung SCC from four tertiary referral hospitals in Taiwan who received second-line treatment initiation between June 2014 and October 2020. The inclusion criteria were as follows: (a) histologically confirmed as locally advanced or metastatic SCC; and (b) disease recurrence after first-line chemotherapy, without prior immunotherapy exposure. Patients who were treated with other epidermal growth factor receptor (EGFR)-TKIs were not included. Data were extracted from patient’s electronic medical records and included demographic and clinical characteristics comprising sex, age, comorbidities, Eastern Cooperative Oncology Group performance status (ECOG PS), initial cancer stage, metastatic sites, first- and second-line treatment, and clinical response.

All the patients included for analysis in the study composed the original cohort, and a propensity-score (PS) approach with 1:1 matching with caliper width of 0.2 was applied to the original cohort to build a propensity-score matching (PSM) cohort. Propensity scores was created through logistic regression as a function of age, sex, first-line chemotherapy, initial clinical stage, baseline ECOG PS, brain metastasis at baseline. We conducted outcome analysis based on second-line treatment for both original cohort and PSM cohort.

The primary endpoint of this study was PFS on second-line treatment, defined as the time from the start of second-line treatment to the time of treatment progression or death, whichever occurred first. The key secondary endpoint was OS, defined as the time from initial lung cancer diagnosis to the time of death. Other secondary endpoints included the objective response rate (ORR), disease control rate (DCR), and treatment-related adverse events (TRAEs). Categorical variables were presented as numbers with percentages and were compared using Fisher’s exact test. Continuous variables were summarized as median with interquartile range (IQR), and compared using the Mann–Whitney U test. Survival analysis was performed using the Kaplan–Meier method. The median and 2-sided 95% confidence intervals (CIs) were determined, and significance was assessed using the log-rank test. Hazard ratios and corresponding 95% CIs were estimated using a Cox proportional-hazards model. As this study involved human participants, all procedures were performed in accordance with the ethical standards of the Institutional and National Research Committee and the 1964 Declaration of Helsinki and its later amendments or comparable ethical standards. This study was subject to the supervision and management of the ethics committees of all participating institutes. Statistical analyses were performed using MedCalc Statistical Software version 19.5.3 (MedCalc Software bvba, Ostend, Belgium) and SPSS software version 25.0 (SPSS Inc., Armonk, NY).

## Results

Overall, 108 patients were identified for inclusion during the study period and formed the original cohort: 19 received afatinib, and 89 received chemotherapy as second-line treatment (Table 1). The median age at initiation of second-line therapy was 71 years (interquartile range [IQR], 63–82 years) in the afatinib group and 67 years (IQR, 61–74 years) in the chemotherapy group. At initial diagnosis, most patients had stage IV disease (afatinib, 63.2%; chemotherapy, 73%), the percentage of current smokers was higher among patients who received afatinib than among those who received chemotherapy (68.4% vs. 49.4%). The contralateral lung was the most common site of metastatic disease (36.8 and 28.1% in the afatinib and chemotherapy groups, respectively), while 6 (31.6%) and 12 (13.5%) patients in the afatinib and chemotherapy groups had brain metastases, respectively. Thirty-six percent of patients in the afatinib group were tested for EGFR mutation, compared to 43.8% of the patients in the chemotherapy group. The expression level of PD-L1 was comparable between two groups. A total of 19 patient pairs were PS matched from the original cohort (PSM cohort), the demographic and clinical characteristics were matched between group.

**Table 1:** Baseline Demographic and Clinical Characteristics

	Original cohort			Propensity-score matching cohort		
Second-Line Treatment	Afatinib (*n* = 19)	Chemotherapy (*n* = 89)	*p*-value	Afatinib (*n* = 19)	Chemotherapy (*n* = 19)	*p*-value
Age, y (IQR)	71 (63–82)	67 (61–74)	0.107	71 (63–82)	66 (60–74)	0.144
Male, n (%)	14 (73.7)	73 (82.0)	0.404	14 (73.7)	16 (84.2)	0.426
Smoking status, n (%)			0.875			0.721
Never smoker	5 (26.3)	25 (28.1)		5 (26.3)	6 (31.6)	
Ex-smoker	1 (5.3)	21(23.6)		1 (5.3)	3 (15.8)	
Current smoker	13 (68.4)	44 (49.4)		13 (68.4)	10 (52.6)	
First-line chemotherapy, n (%)			0.118			0.174
Platinum-based	11 (57.9)	71 (79.8)		11 (57.9)	15 (78.9)	
Not platinum-based	8 (42.1)	18 (20.2)		8 (42.1)	4 (21.1)	
Initial clinical stage, n (%)			0.331			0.283
IIIB/C	7 (36.8)	24 (27.0)		7 (36.8)	4 (21.1)	
IV	12 (63.2)	65 (73.0)		12 (63.2)	15 (78.9)	
Baseline ECOG PS			0.135			0.221
0/1	12 (63.2)	76 (85.4)		12 (63.2)	16 (84.2)	
≥ 2	7 (36.8)	13 (14.6)		7 (36.8)	3 (15.8)	
EGFR status, n (%)			0.577			0.560
Wild-type	7 (36.8)	39 (43.8)		7 (36.8)	5 (26.3)	
Not tested	12 (63.2)	50 (56.2)		12 (63.2)	14 (73.7)	
PD-L1 expression level, n (%)			0.278			0.530
< 1%	5 (26.3)	13 (14.6)		5 (26.3)	2 (10.5)	
1–49%	2 (10.5)	27 (30.3)		2 (10.5)	3 (15.8)	
> 50%	2 (10.5)	10 (11.2)		2 (10.5)	1 (5.3)	
Not tested	10 (52.6)	39 (43.8)		10 (52.6)	13 (68.4)	
Sites of metastatic disease, n (%)						
Liver	0 (0)	10 (11.2)	0.125	0 (0)	1 (5.3)	0.311
Contralateral lung nodule	7 (36.8)	25 (28.1)	0.448	7 (36.8)	3 (15.8)	0.141
Bone	3 (15.8)	25 (28.1)	0.267	3 (15.8)	5 (26.3)	0.426
Pleural (nodules, effusion)	2 (10.5)	15 (16.9)	0.641	2 (10.5)	5 (26.3)	0.209
Brain	6 (31.6)	12 (13.5)	0.055	6 (31.6)	4 (21.1)	0.521
Other	0 (0)	7 (7.9)	0.718	0 (0)	3 (15.8)	0.107
Comorbidity, n (%)						
Hypertension	4 (21.1)	42 (47.2)	0.054	4 (21.1)	9 (47.4)	0.087
Chronic kidney disease	0 (0)	3 (3.4)	0.417	0 (0)	1 (5.3)	0.311
Diabetes mellitus	4 (21.1)	22 (24.7)	0.734	4 (21.1)	5 (26.3)	0.703
Hepatic disease	0 (0)	9 (10.1)	0.148	0 (0)	2 (10.5)	0.214
Pulmonary disease	4 (21.1)	25 (28.1)	0.734	4 (21.1)	2 (10.5)	0.374

*IQR* interquartile range; *ECOG PS* Eastern Cooperative Oncology Group performance status; *EGFR* epidermal growth factor receptor; *PD-L1* programmed cell death protein 1

Among the chemotherapy group, 33.7% of patients received gemcitabine or gemcitabine-based therapy; 28.1% received vinorelbine or vinorelbine-based therapy; 28.1% received docetaxel or docetaxel-based therapy; 7.9% received other regimens; and 2.2% received atezolizumab monotherapy (Table 2). Median time on treatment for first-line chemotherapy was 6.0 months [95% CI, 1.2–9.1 months] in the afatinib group and 5.4 months [95% CI, 0.9–11.2 months] in the chemotherapy group (Table 3).

**Table 2:** Second-Line Treatment Regimens in the Chemotherapy Group (*n* = 89)

Second-Line Therapy Received, n (%)	
Gemcitabine	28 (31.5)
Vinorelbine	24 (26.9)
Docetaxel	22 (24.7)
Others	7 (7.9)
Carboplatin/gemcitabine	2 (2.2)
Atezolizumab	2 (2.2)
Carboplatin/docetaxel	2 (2.2)
Carboplatin/vinorelbine	1 (1.1)
Pembrolizumab/docetaxel	1 (1.1)

**Table 3:** Best Overall Tumor Response to Second-Line Treatment

	Original cohort			Propensity-score matching cohort		
	Afatinib (*n* = 19)	Chemotherapy (*n* = 89)	*p*-value	Afatinib (*n* = 19)	Chemotherapy (*n* = 19)	*p*-value
Disease control, n (%)	16 (84.2)	41 (46.1)	0.003*	16 (84.2)	8 (42.1)	0.007*
Objective response, n (%)	5 (26.3)	7 (7.9)	0.020*	5 (26.3)	2 (10.5)	0.209*
CR, n (%)	0 (0)	0 (0)	..	0 (0)	0 (0)	..
PR, n (%)	5 (26.3)	7 (7.9)	..	5 (26.3)	2 (10.5)	..
SD, n (%)	11 (57.9)	34 (38.2)	..	11 (57.9)	6 (31.6)	..
PD, n (%)	2 (10.5)	43 (48.3)	..	2 (10.5)	7 (36.8)	..
NE, n (%)	1 (5.3)	5 (5.6)	..	1 (5.3)	2 (10.5)	..
Time on first-line treatment, months (95% CI)	6 (1.2–9.1)	5.4 (0.9–11.2)	..	6 (1.2–9.1)	3.5 (0.4–13.9)	..
PFS, months (95% CI)	4.7 (0.1–7.5)	2.6 (0.9–6.7)	..	4.7 (0.1–7.5)	1.9 (0.4–8.4)	..
OS, months (95% CI)	16.0 (6.1–22.0)	12.3 (6.2–33.9)	..	16.0 (6.1–22.0)	9.9 (6.2–39.4)	..

*CR* complete response; *PR* partial response; *SD* stable disease; *PR* progressive disease; *NE* not evaluable; *PFS* progression-free survival of second-line treatment; *OS* overall survival

*Categorical variables were compared using the Fisher’s exact test

**Fig. 1:**
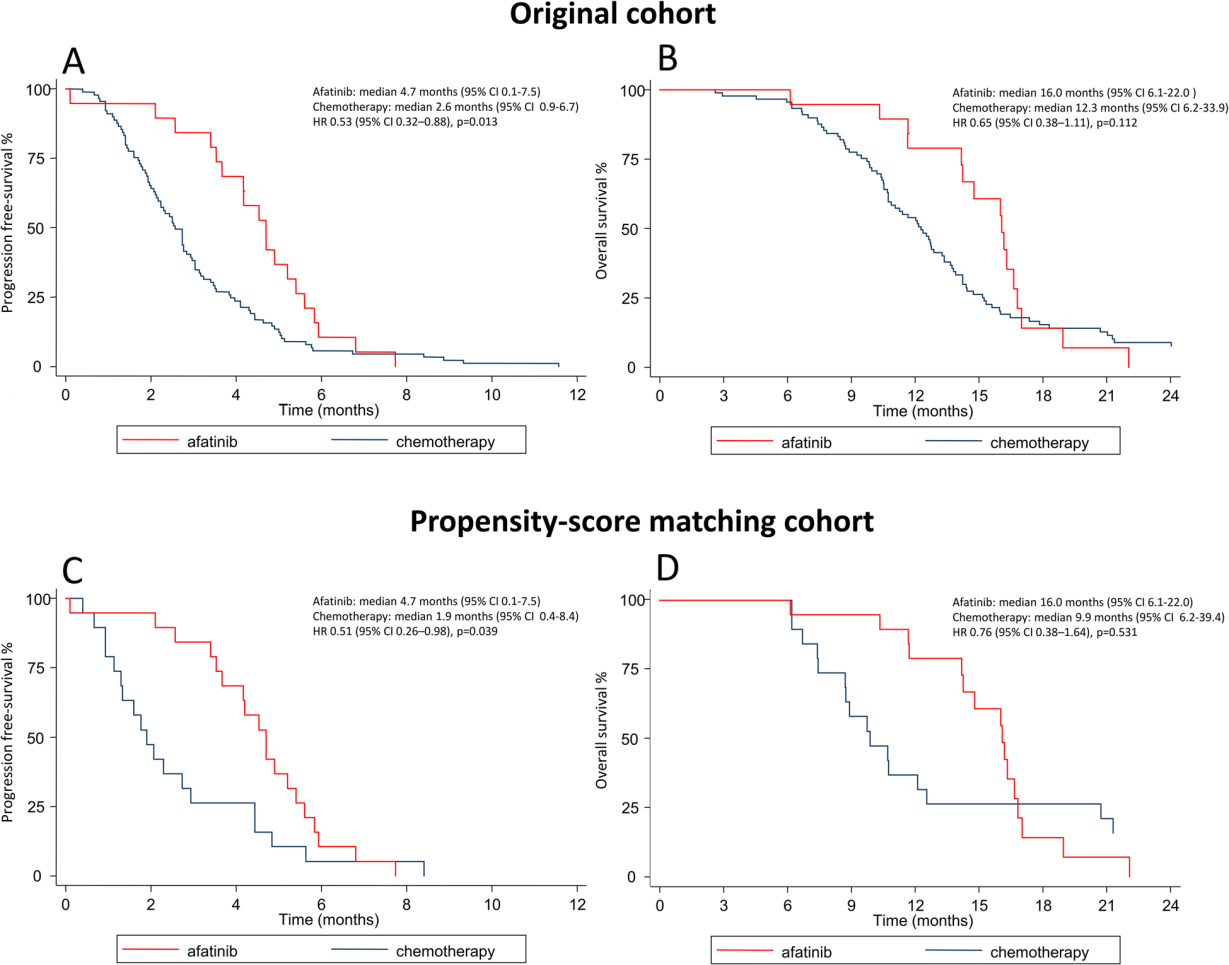
Progression-free survival (Panel **A**) and overall survival (Panel **B**) of the original cohort; Progression-free survival (Panel **C**) and overall survival (Panel **D**) of the propensity-score matching cohort. Abbreviations: HR = hazard ratio; CI = confidence interval

After a median follow-up of 12.7 months (IQR, 10.1–16.0 months), PFS was significantly longer among patients who received afatinib than among those who received chemotherapy (median 4.7 months [95% CI, 0.1–7.5 months] vs. 2.6 months [95% CI, 0.9–6.7 months]; hazard ratio (HR) 0.53 [95% CI, 0.32–0.88], *p* = 0.013; Fig. 1A). More patients in the afatinib group than in the chemotherapy group had an objective response (ORR:26.3% vs. 7.9%, *p* = 0.020), according to an independent review (Table 3). Disease control was also greater in the afatinib group than in the chemotherapy group (DCR:84.2% vs. 46.1%, *p* = 0.003). OS was comparable between groups (median 16.0 months among afatinib group [95% CI, 6.1–22.0 months] vs. 12.3 months among chemotherapy group [95% CI, 6.2–33.9 months]; HR 0.65 [95% CI, 0.38–1.11], *p* = 0.112; Fig. 1B). Pertaining to the PSM cohort, results of outcomes analysis were highly consistent with the original cohort. PFS was significantly longer in patients who received afatinib than those who received chemotherapy (median 4.7 months [95% CI, 0.1–7.5 months] vs. 1.9 months [95% CI, 0.4–8.4 months]; hazard ratio (HR) 0.51 [95% CI, 0.26–0.98], *p* = 0.039; Fig. 1C). OS was comparable between groups (median 16.0 months among afatinib group [95% CI, 6.1–22.0 months] vs. 9.9 months among chemotherapy group [95% CI, 6.2–39.4 months]; HR 0.76 [95% CI, 0.38–1.64], *p* = 0.531; Fig. 1D). The ORR and DCR among patients treated with afatinib surpassed those of patients treated with chemotherapy (Table 3).

Beyond second-line treatment, twenty-three patients (3 patients in the afatinib group and 20 patients in the chemotherapy group) accepted further third-line treatment. All three patients from afatinib group received chemotherapy alone or in combination with anti-angiogenesis agents. Among the chemotherapy group, thirteen patients received chemotherapy alone or in combination with anti-angiogenesis agents, three received immunochemotherapy, one received immunotherapy alone and the other three received EGFR-TKI as third-line treatment (supplementary Table 1).

When examining TRAEs, 89.5% of patients in the afatinib group versus 73.0% in the chemotherapy group reported any grade of adverse effects. The majority of patients (89.5%) in the afatinib group experienced skin (paronychia, rashes, or acne) and gastrointestinal (nausea, vomiting, or diarrhea) toxicities; less frequent events included mucositis (21.1%) and laboratory-confirmed hematologic toxicity (5.3% presented with neutropenia, anemia, or thrombocytopenia, which developed after treatment initiation). By contrast, hematologic toxicity and neuropathy were more prominent in the chemotherapy group (68.5 and 16.9%, respectively); skin and gastrointestinal toxicities were less commonly reported among patients who received chemotherapy (Table 4).

**Table 4:** Any Grade of Treatment-Related Adverse Events (TRAEs)

	Afatinib (*n* = 19)	Chemotherapy (*n* = 89)	*p*-value
TRAEs	17 (89.5)	65 (73.0)	0.128
Skin toxicity (paronychia, rash, acne), n (%)	17 (89.5)	5 (5.6)	< 0.001
Gastrointestinal toxicity (nausea, vomiting, diarrhea), n (%)	17 (89.5)	10 (11.2)	< 0.001
Mucositis, n (%)	4 (21.1)	10 (11.2)	0.248
Abnormal liver function, n (%)	0 (0)	7 (7.9)	0.206
Hematologic toxicity (neutropenia, anemia, thrombocytopenia), n (%)	1 (5.3)	61 (68.5)	< 0.001
Neuropathy, n (%)	0 (0)	15 (16.9)	0.156

## Discussion

The results of this study provide insights regarding the efficacy and safety of second-line afatinib for the treatment of patients with lung SCC following disease progression after first-line chemotherapy. In the present study, the median PFS with second-line afatinib (4.7 months) was significantly longer than that with second-line chemotherapy (2.6 months). Although the OS was longer in the afatinib group (16.0 months) than in the chemotherapy group (12.3 months), this difference did not reach significance. The PSM cohort carried the consistent consequence, improved the convincing power and indicated our findings were robust. Our results implied that afatinib represents an optimal treatment choice following chemotherapy in patients with lung SCC following progression after first-line treatment in a real-world setting.

The introduction of ICIs for the treatment of advanced lung SCC represents a breakthrough associated with improved outcomes over the past decade. First-line ICI monotherapy or ICI combination therapy is supported by several large Phase III studies, which have demonstrated remarkably extended survival [13, 14, 19, 20]. Notably, combined immunochemotherapy also provided longer PFS and probable longer OS than chemotherapy alone in patients with metastatic SCC NSCLC, regardless of PD-L1 level [10, 21]. ICIs have practically replaced docetaxel as the second-line treatment for advanced NSCLC, several ICIs demonstrated significantly longer OS than docetaxel, regarding to different PD-L1 expression level [22–29]. However, ICIs are not always available or affordable in certain countries, including Taiwan; therefore, we did not include patients treated with ICIs in this study (ICI-naïve patients only). In addition, among patients who present with contraindications to immunotherapy, such as autoimmune diseases or previous solid organ transplant, combination cytotoxic chemotherapy or alternative afatinib (for patients unfit for chemotherapy) is recommended [30, 31]. We suggest that afatinib represents a second-line alternative treatment option for immunotherapy unfit advanced lung SCC patients.

The role of EGFR-TKIs in the treatment of lung SCC without EGFR-activating mutations has been evaluated in several clinical trials. The BR.21 trial, a multicentric, randomized, Phase III trial, reported a median PFS of 2.2 months in the erlotinib group versus 1.8 months in the placebo supportive care group (HR, 0.61, *p* < 0.001) [32]. By contrast, the TAILOR and DELTA trials further compared erlotinib against the standard second-line chemotherapy treatment with docetaxel in pretreated patients with advanced NSCLC unselected for EGFR mutation. The OS and PFS among patients treated with docetaxel surpassed those of patients treated with erlotinib, which was especially evident in the subgroup with SCC histology [33, 34]. Therefore, the FDA restricted erlotinib second-line indications to patients with EGFR del 19 or L858R mutations in 2016 [35]. The therapeutic role of afatinib was established by the LUX-Lung 8 study, a large, multi-national, Phase III trial specifically designed for a population in which EGFR mutations are almost absent. The study reported an advantage for both the primary endpoint of PFS (0.5 months) and the secondary endpoints of OS (1.1 months), DCR, and symptom control. Although EGFR-TKIs showed better tolerability and comparable OS as second-line therapy compared with chemotherapy in a meta-analysis [36], the results were primarily due to the comparison between erlotinib and chemotherapy. The direct comparison between afatinib and chemotherapy for second-line treatment was lacking. To the best of our knowledge, this retrospective study provides the first available insights into the real-world use of afatinib compared with chemotherapy following the failure of first-line chemotherapy in patients with lung SCC.

The Phase III trial REVEL compared the combination of docetaxel plus ramucirumab against docetaxel alone in patients with SCC and non-SCC NSCLC who previously received platinum-based treatment, which revealed significant improvement in PFS (4.5 vs. 3.0 months; HR, 0.76, *p* < 0.0001) and OS (10.5 vs. 9.1 months; HR, 0.86, *p* = 0.02) for the combination group. No patients in our study received vascular endothelial growth factor receptor (VEGFR) monoclonal antibodies in combination with docetaxel, which may lower the treatment effect in the chemotherapy group. However, among patients 65 years or older, the additional benefits of ramucirumab were not as evident as among younger patients, and extra toxicity may occur [37, 38]. The patients in our study were older than those included in previous major clinical trials; thus, the benefits and tolerance for combination treatment should be considered thoroughly according to the patients’ physiological condition. Most patients in the present study encountered manageable skin and gastrointestinal toxicities; laboratory-confirmed hematologic toxicity or neuropathy occurred less frequently in afatinib-treated patients compared with chemotherapy-treated patients. Differences in the presentation of adverse effects implied that second-line afatinib treatment for lung SCC offered better tolerability and quality of life than chemotherapy, which may lead to longer survival.

Previously approved agents have been subsequently moved more marginally in the therapeutic algorithm for SCC because of the approval of ICIs as first- and second-line treatments. However, EGFR-TKIs represent an available therapeutic option for lung SCC following the failure of previous lines of treatment, and EGFR-TKIs are easily managed as an oral drug with good tolerability compared with chemotherapy [39, 40]. Moreover, an applicable percentage of patients gained modest improvement in symptom control and quality of life from afatinib use [41]. Among patients who are not suitable candidates for cytotoxic chemotherapy or immunotherapy, afatinib may represent a convenient second- or third-line treatment option. Afatinib has been proved to be a good immuno-modulator which might modify the microenvironment of tumors, upregulate the PD-L1 expression, and possibly improved the immunotherapy effect [42, 43]. Further investigation of the crosstalk between the EGFR and PD-L1 pathways may be a rational approach to improve lung squamous cell carcinoma treatment [44]. Sequential or Combined immunotherapy with various of EGFR-TKI did show some encouraging antitumor activity but the safety profiles were highly varied [45–47]. Studies of second-line or third-line afatinib after chemotherapy or immunotherapy is ongoing currently. Only a minority of the patients received third-line treatment in this study, which didn’t aim to answer the complexities of the crosstalk between these pathophysiology pathways; nevertheless, we presented a convincing result that second-line afatinib may be considered as alternative treatment choice for first-line immunotherapy unfit advanced lung SCC patients.

This study has several limitations. First, due to the retrospective nature of the study, the data for some patients might be influenced by incomplete records and uneven patient characteristics. Patients with good ECOG PS were more likely to receive first-line platinum-based chemotherapy, and younger patients represented a higher proportion of the chemotherapy group. By contrast, afatinib was more commonly administered for patients with poor PS and among older individuals, complementing the PFS and OS benefits of afatinib. Second, this study recruited patients from only four institutes, and the limited provider and patient source may result in a highly selected patient population. Although we conducted a multi-institute study, the number of patients who received second-line afatinib treatment was small, reflecting the dramatic decrease in the frequency of using targeted therapy as second-line treatment during the immunotherapy era. PS matching analysis was used to eliminate of potential confounding, which indicated that our findings were robust and increased the convincing power of the study. Therefore, the results of our study were commendable and may offer a reference for a clinical setting in which immunotherapy is not universally available. Third, detailed subgroup analysis was difficult to be executed owing to the limited sample size. In order to avoid false positives due to multiple comparisons, this study didn’t report subgroup analysis to identify which specific patient group might benefit most from afatinib. Finally, the majority of the patient did not receive EGFR mutation test and further genomic studies, specific information regarding driver oncogenes detection was not available, and the underlying biology and the nature of progression in individual patients might have influenced the treatment effect. Further large-scale prospective studies are warranted to validate these findings.

## Conclusions

Afatinib offered longer PFS and comparable OS to chemotherapy in patients with lung SCC progression after first-line chemotherapy in a real-world setting. At present, afatinib is the only approved oral agent following chemotherapy in patients with lung SCC, and physicians are encouraged to consider it as a second-line alternative treatment choice for immunotherapy unfit advanced lung SCC patients.

## Supplementary Information

**Additional file 1: Supplementary Table 1.** 3rd Line Treatment Regimens (*n* = 23).

## Data Availability

The datasets used and/or analyzed during the current study are available from the corresponding author on reasonable request.

## Abbreviations

NSCLC: Non-small-cell lung cancer
SCC: Squamous cell carcinoma
EGFR: Epidermal growth factor receptor
ALK: Anaplastic lymphoma kinase
ROS1: C-ros oncogene 1
BRAF: Murine sarcoma viral oncogene homolog B
ICIs: Immune checkpoint inhibitors
PD-L1: Programmed death-ligand 1
TPS: Tumor proportion score
PFS: Progression-free survival
OS: Overall survival
ESMO: European Society of Medical Oncology
EGFR-TKI: EGFR-tyrosine kinase inhibitor.
ECOG PS: Eastern Cooperative Oncology Group performance status
ORR: Objective response rate
DCR: Disease control rate
HR: Hazard ratio.
TRAEs: Treatment-related adverse events
VEGFR: Vascular endothelial growth factor receptor.
