# Supplementary material for: Real-world effectiveness of second-line Afatinib versus chemotherapy for the treatment of advanced lung squamous cell carcinoma in immunotherapy-naïve patients
Source: BMC Cancer. 2021 Nov 15;21:1225. doi: 10.1186/s12885-021-08920-3 (PMC8594240; doi:10.1186/s12885-021-08920-3)
Supplement: Supplementary file 1 — Additional file 1: Supplementary Table 1. 3rd Line Treatment Regimens (n = 23). [file 12885_2021_8920_MOESM1_ESM.docx]

**Supplementary table 1. The Third Line Treatment Regimens (n = 23)**

| 3^rd^ Line Therapy Received | Afatinib group  (n=3) | Chemotherapy group (n=20) |
| --- | --- | --- |
| Chemotherapy +/- anti-angiogenesis agents | 3 | 13 |
| Immunochemotherapy | 0 | 3 |
| Immunotherapy alone | 0 | 1 |
| EGFR-TKI | 0 | 3 |
